# A comparative study of chest CT findings regarding the effects of regional dust exposure on patients with COPD living in urban areas and rural areas near cement plants

**DOI:** 10.1186/s12931-021-01649-4

**Published:** 2021-02-06

**Authors:** Junghyun Kim, Bom Kim, So Hyeon Bak, Yeon-Mok Oh, Woo Jin Kim

**Affiliations:** 1 Division of Pulmonary and Critical Care Medicine, Department of Internal Medicine, National Medical Center, Seoul, Republic of Korea; 2 Department of Internal Medicine and Environmental Health Center, Kangwon National University, Chuncheon, Republic of Korea; 3 Deparment of Radiology, School of Medicine, Kangwon National University, Chuncheon, Republic of Korea; 4 Department of Pulmonary and Critical Care Medicine and Clinical Research Center for Chronic Obstructive Airway Diseases, Asan Medical Center, University of Ulsan College of Medicine, Seoul, Republic of Korea

**Keywords:** COPD, CT, Dust, Emphysema

## Abstract

**Background:**

The clinical and radiological presentation of chronic obstructive pulmonary disease (COPD) is heterogenous depending on the characterized sources of inflammation. This study aimed to evaluate COPD phenotypes associated with specific dust exposure.

**Methods:**

This study was designed to compare the characteristics, clinical outcomes and radiological findings between two prospective COPD cohorts representing two distinguishing regions in the Republic of Korea; COPD in Dusty Area (CODA) and the Korean Obstructive Lung Disease (KOLD) cohort. A total of 733 participants (n = 186 for CODA, and n = 547 for KOLD) were included finally. A multivariate analysis to compare lung function and computed tomography (CT) measurements of both cohort studies after adjusting for age, sex, education, body mass index, smoking status, and pack-year, Charlson comorbidity index, and frequency of exacerbation were performed by entering the level of FEV1(%), biomass exposure and COPD medication into the model in stepwise.

**Results:**

The mean wall area (MWA, %) became significantly lower in COPD patients in KOLD from urban and metropolitan area than those in CODA cohort from cement dust area (mean ± standard deviation [SD]; 70.2 ± 1.21% in CODA vs. 66.8 ± 0.88% in KOLD, p = 0.028) after including FEV1 in the model. COPD subjects in KOLD cohort had higher CT-emphysema index (EI, 6.07 ± 3.06 in CODA vs. 20.0 ± 2.21 in KOLD, p < 0.001, respectively). The difference in the EI (%) was consistently significant even after further adjustment of FEV1 (6.12 ± 2.88% in CODA vs. 17.3 ± 2.10% in KOLD, p = 0.002, respectively). However, there was no difference in the ratio of mean lung density (MLD) between the two cohorts (p = 0.077). Additional adjustment for biomass parameters and medication for COPD did not alter the statistical significance after entering into the analysis with COPD medication.

**Conclusions:**

Higher MWA and lower EI were observed in COPD patients from the region with dust exposure. These results suggest that the imaging phenotype of COPD is influenced by specific environmental exposure.

## Introduction

Chronic obstructive pulmonary disease (COPD) is defined as progressive airway obstruction with parenchymal lung destruction and airway inflammation. The prevalence of COPD is increasing and COPD is predicted to become the 3rd leading cause of death worldwide by 2030 [1, 2]. The association of COPD with poor clinical outcomes including lung function decline, acute exacerbations (AE) and mortality has suggested clues for further studies to identify specific phenotypes and modifiable risk factors for COPD in individual daily life.

The clinical and radiological presentations of COPD have proposed heterogeneity. Inflammation, characterized by COPD, can differ by smoking, air pollutants, biomass, or genetic factors leading to diverse clinical or radiological findings. Heterogeneity of COPD on computed tomography (CT) findings have been broadly characterized as either; emphysema-predominant or airway-predominant type [3, 4]. Bronchial parameters such as bronchial lumen area (LA) and bronchial wall area (WA) represented bronchial wall thickness and have been shown to correlate with spirometric parameters [5–7]. The mean value of two segmental bronchi was defined as mean wall area (MWA) [8]. The values of airway thickness such as MWA% may be related to inflammation of airway and are associated with symptom of chronic bronchitis, acute exacerbation and bronchodilator responsiveness. Increased MWA% has strong associations with decreased lung function and increased frequency of acute exacerbations [9]. Phenotypic differences have been reported in the studies of COPD investigating the association between biomass and smoke exposure, which were suggest a small airway disease rather than an emphysematous phenotype upon cigarette smoking exposure [10–12]. A cluster analysis of four subgroups of smokers suggested a genetic effect with an association between the clinical characteristics of COPD and known COPD-associated genetic variants [13].

To evaluate COPD phenotypes for specific exposure, it is necessary to compare radiologic findings between patients who reside in regions representing specific dust exposure and non-residents. In this study, we investigated whether there is a difference in structural findings in CT by evaluating the characteristic of patients with COPD who lived in two functional areas; urban areas where mainly composed of metropolitan regions with residential areas, without large industrial emissions and rural areas, where large cement plants are located nearby expecting exposure to different composition of dust.

## Methods

### Study design and subjects

This study was designed to compare characteristics and clinical outcomes using radiological findings between two prospective COPD cohorts representing distinguishing regions in the Republic of Korea; COPD in Dusty Area (CODA) and the Korean Obstructive Lung Disease (KOLD) cohorts. The CODA cohort was prospectively recruited from 2012 to 2015 to observe the clinical outcomes of Korean COPD patients from rural area living near cement plants [14]. This cohort was enrolled from Kangwon and Chungbuk provinces in Korea where cement plants were mostly located. The KOLD cohort, which comprised participants from 17 centers recruited from 2005 to 2013, enrolled patients at 17 centers in an urban area in Korea [15]. COPD subjects in both cohorts were recruited for medical examination which consisted of a questionnaire regarding environmental exposure, health-related symptoms and behaviors, and laboratory findings with a pulmonary function test (PFT) with post-bronchodilator use and CT. Among participants in both cohorts, individuals with COPD defined according to European Respiratory Society/American Thoracic Society (ERS/ATS) guidelines (post-bronchodilatorforced expiratory volume in 1 s (FEV1)/Forced vital capacity (FVC) < 70%) and having a history of smoking of over 10 pack-years (PY) were eligible. Finally, 733 participants (n = 186 for CODA, and n = 547 for KOLD, for each) were included.

The study design and methods were approved by the Institutional Review Board (IRB) of Kangwon National University Hospital (IRB No. KNUH 2012-06-007) and the study was conducted following the tenets of the Declaration of Helsinki.

### Spirometry and biomass exposure

Spirometry tests were performed using standardized equipment by qualified technicians following the ATS/ERS guidelines [16]. Spirometry tests were repeated at least three times to achieve within- and between-maneuver acceptability criteria. After the initial spirometry testing (pre-dose spirometry), a dose of 100 μg of salbutamol was fully inhaled in one breath, and the breath was then held for 5 to 10 s before exhalation. Two separate doses (total dose 200 μg) were administered at approximately 30-s intervals. Three additional spirometry tests were performed between 10 and 15 min later for reversibility testing. Spirometric tests were done by identical ways in both cohorts. The predicted value of spirometry were calculated by Choi’ reference equation which was validated for Korean population [17].

In this study, we asked previous biomass exposure which was also known to influence the phenotype of COPD by questionnaire [10]. The question for direct exposure to charcoal and wood, which were the most common fuel for cooking and heating in Korea until 1980s regardless of where persons lived, were asked as follow; “Have you ever been exposed to wood or charcoal fuels for cooking and/or heating?”. The presence of biomass exposure was defined by more than 10 years of direct exposure history to charcoal and wood.

### Computed tomography

Volumetric CT scans were taken at full inspiration and expiration, using a first-generation dual-source CT system (Somatom Definition, Siemens Healthcare, Forchheim, Germany) in CODA and 16-multidetector CT scanners produced by three different manufacturers (Somatom Sensation 16; Siemens Medical Systems, Bonn, Germany; GE Lightspeed Ultra; General Electric Healthcare, Milwaukee, WI, USA; and Philips Brilliance 16, Philips Medical Systems, Best, Netherlands) in KOLD based on their reports of both the CODA and KOLD studies [15]. Scan parameters were as follows: 120 or 140 kVp; 100–135 effective mA with dose modulation; slice thickness and reconstruction intervals of 0.6–0.8 mm. Detailed information regarding the measurement of CT findings has been previously described and an identical protocol for CT measurement were applied to participants of both cohorts [5, 6, 8]. Whole-lung images were extracted from the chest wall, mediastinum and large airway automatically, and the attenuation coefficient of each pixel was calculated. The mean of the lung density values derived from the histogram of whole lung was defined as mean lung density (MLD). From the CT data, the emphysema index (EI) was defined using the volume fraction (%) of the lung below − 950 Hounsfield Units (HU) at maximum inspiration. The ratio MLD at full expiration and inspiration was automatically calculated. The measured values are the mean of density of air in alveoli and the surrounding epithelial, capillary and extracellular matrix and small airways. The airway dimensions, wall area (WA), lumen area (LA), and WA percent (i.e., WA/(WA + LA) × 100) were measured near the origin of the two segmented bronchi (right apical and left apico-posterior) to assess airway thickness using in-house software. The mean value of two segmental bronchi was defined as mean wall area (MWA) [8]. To express the morphologic characteristics quantitatively, we used the most commonly employed approach: EI as emphysema extent, the ratio of the MLD on expiration and inspiration as air trapping extent and the percentage of the MWA (MWA %) as the extent of airway disease.

### Statistical analysis

The baseline characteristics of the patients were reported as mean ± standard deviation (SD). The distribution of each variable was tested by Kolmogorov–Smirnov (K–S) and Shapiro–Wilk test. Normal distribution parameters were analyzed using an independent sample t-test, and non-normal distribution data, such as the EI and hs-CRP, were analyzed using the Mann–Whitney U-test. As EI was not in normal distribution, it was analyzed by log-transformed value. Multivariate analysis of the means of clinical and CT variables were estimated using the least squares mean of the generalized linear model. Age, body mass index (BMI), and post-bronchodilator FEV1 (% of predicted) were included in the model in stepwise as these variables were associated with the extent of emphysema in smokers with COPD [18, 19]. In addition, a past- biomass exposure was also entered into multivariate analysis [10]. All statistical analyses were performed using the SAS statistical package, Version 9.2 (SAS Institute Inc., Cary, NC, USA), with p-values of less than 0.05 considered as statistically significant.

## Results

Table 1 shows the baseline characteristics of the study population in the CODA and KOLD cohort studies. Participants in CODA were older than those in the KOLD cohort (mean ± SD; 72.5 ± 7.1 vs. 67.4 ± 8.0, p < 0.001). Most of the participants are male (98.9% in CODA and 96.9% in KOLD) and former smokers (64.0% in CODA and 64.7% in KOLD, respectively) in both cohorts. Pack-years (PYs) of smoking were higher in participants from KOLD (46.4 ± 25.8 PYs) than participants from CODA (34.4 ± 24.7 PYs) despite there was no significant difference in current smoking status. Participants in the CODA cohort had more comorbidities (Charlson Comorbidity Index [CCI] ≥ 2: 18.3% in CODA vs. 6.9% in KOLD, p < 0.001)) and fewer severe COPD exacerbations requiring hospitalization over the past year (p < 0.001). FEV1 was higher in CODA. The FVC was higher in participants from the KOLD cohort. Any history of biomass exposure was higher in KOLD participants (74.6% in KOLD vs. 64.0% in CODA, p = 0.006). The CT measurements between the two cohorts were significantly different. Participants in CODA cohorts showed higher MWA (68.58 ± 4.79 vs. 66.05 ± 4.95, p < 0.001) and KOLD participants showed higher EI than those in CODA (9.28 ± 7.75 vs. 19.64 ± 14.55, p < 0.001), respectively.

**Table 1:** General characteristics of participants in CODA and KOLD cohort studies

	CODA (n = 186)		KOLD (n = 547)			
	N	Mean ± SD or N (%)	N	Mean ± SD or N (%)	*P*	log-formed P P for log-transformed value
Age (year)	186	72.5 ± 7.1	547	67.4 ± 8.0	< .0001	
40 ~ 59		8 (4.3)		90 (16.5)	< .0001	
60 ~ 69		50 (26.9)		232 (42.4)		
70 ~ 79		100 (53.8)		195 (35.6)		
80 ~ 96		28 (15.1)		30 (5.5)		
Gender (N, %)						
Male		184 (98.9)		530 (96.9)	0.1318	
Female		2 (1.1)		17 (3.1)		
Education						
< Elementary school		41 (22.0)		45 (9.2)	< .0001	
Elementary school		84 (45.2)		91 (18.6)		
Middle school		28 (15.1)		109 (22.3)		
≥ High school		33 (17.7)		243 (49.8)		
Height (cm)		163.0 ± 6.3		165.6 ± 6.4	< .0001	
Weight (kg)		60.2 ± 10.1		63.2 ± 9.9	0.0004	
BMI (kg/m^2^)		22.6 ± 3.2		23.0 ± 3.1	0.1491	
< 23.0		100 (53.8)		263 (48.2)	0.3776	
23.0 ~ 24.9		44 (23.7)		153 (28.0)		
≥ 25.0		42 (22.6)		130 (23.8)		
Smoking (yes)						
Current		67 (36.0)		193 (35.3)	0.8558	
Former		119 (64.0)		354 (64.7)		
Pack-year of smoking		34.4 ± 24.7		46.4 ± 25.8	< .0001	
History of chronic bronchitis (N, %)						
No		154 (93.3)		427 (89.1)	0.1182	
Yes		11 (6.7)		52 (10.9)		
Charlson comorbidity index (N, %)						
0		107 (57.5)		362 (75.6)	< .0001	
1		45 (24.2)		84 (17.5)		
≥ 2		34 (18.3)		33 (6.9)		
Hospitalized exacerbations in the prior year (N, %)						
0		175 (94.1)		403 (81.1)	0.0001	
1		4 (2.2)		45 (9.1)		
≥ 2		7 (3.8)		49 (9.9)		
Hospitalized exacerbations in the prior year (N, %)						
No		175 (94.1)		403 (80.0)	< .0001	
Yes		11 (5.9)		101 (20.0)		
COPD medications^*^(N, %)						
No		129 (69.4)		163 (36.6)	< .0001	
Yes		57 (30.6)		282 (63.4)		
History of biomass exposure (N, %)						
No		67 (36.0)		123 (25.4)	0.0061	
Yes		119 (64.0)		362 (74.6)		
CAT score	186	17.10 ± 9.50	219	12.20 ± 7.80	< .0001	
Pulmonary function test						
Pre-bronchodilator FEV1 (L)	186	1.79 ± 0.54	546	1.58 ± 0.58	< .0001	
Pre-bronchodilator FVC (L)	186	3.01 ± 0.75	546	3.37 ± 0.81	< .0001	
Post-bronchodilator FEV1 (L)	186	1.89 ± 0.52	547	1.72 ± 0.59	0.0004	
Post-bronchodilator FVC (L)	186	3.22 ± 0.72	547	3.55 ± 0.79	< .0001	
Post-bronchodilator FEV1/FVC	186	0.58 ± 0.09	547	0.48 ± 0.11	< .0001	
BDR, bronchodilator response	186	7.47 ± 13.49	546	10.23 ± 10.45	0.0116	
Chest CT measurement						
Mean wall area (%)	181	68.58 ± 4.79	516	66.05 ± 4.95	< .0001	< .001
Emphysema index	182	9.28 ± 7.75	516	19.64 ± 14.55	< .0001	< .001
Mean lung density	99	0.95 ± 0.04	516	0.94 ± 0.04	0.1075	0.116
Biomarkers						
WBC (× 10^3^/μl)	185	6.95 ± 1.85	448	7.31 ± 2.02	0.0394	0.028
RBC (× 10^6^/μl)	185	4.54 ± 0.47	449	4.70 ± 0.45	< .0001	< .001
Hemoglobin (g/dL)	185	14.32 ± 1.48	451	14.74 ± 1.38	0.0008	0.001
Hematocrit (g/dL)	185	41.74 ± 4.04	449	43.80 ± 3.86	< .0001	< .001
Eosinophil (%)	185	2.98 ± 3.45	443	3.67 ± 3.24	0.0185	0.012
Lymphocyte (%)	185	30.80 ± 8.03	443	31.19 ± 8.67	0.5988	0.932
Neutrophil (%)	185	57.74 ± 8.97	439	57.16 ± 9.64	0.4894	0.411
Neutrophil/lymphocyte	185	2.11 ± 1.00	439	2.21 ± 2.22	0.4242	0.744
hs-CRP (mg/dL)	169	0.29 ± 0.59	299	0.39 ± 1.01	0.1630	< .001

Data are presented as mean ± standard deviation for continuous variables or number (%) for categorical variables

Statistical differences between the two groups were estimated by independent sample t-test for parametric values and Mann–Whitney U-test for non-parametric values after log-transformation, or chi-square test for categorical variables

Subject criterion: COPD (post FEV1/FVC < 0.7) and pack-year ≥ 10

BDR was calculated as follows: FEV1(L) post-bronchodilator minus FEV1(L) pre-bronchodilator / FEV1(L) pre-bronchodilator × 100%

*BMI* body mass index, *CT* computed tomography, *BDR* bronchodilator response, *FEV1* forced expiratory volume in 1 s, *FVC* forced vital capacity, *CAT* COPD assessment test, *WBC* white blood cell, *RBC* red blood cell, *hs-CRP* high-sensitivity c-reactive protein

^*^COPD medication includes LAMA, LABA, SABA, or ICS/LABA

Multivariate analysis for the comparison of lung function and CT measurements of both cohorts after adjusting for age, gender, educational attainment, BMI, smoking status, pack-years of their smoking among smokers, CCI, and COPD exacerbation showed higher EI in KOLD cohorts than in the CODA cohort (6.07 ± 3.06, in CODA vs. 20.0 ± 2.21 in KOLD, p < 0.001). This significant difference was consistent even after further adjustment for FEV1 (6.12 ± 2.88 vs. 17.3 ± 2.10, respectively) (Table 2). The MWA was also significantly lower in the KOLD cohort (70.2 ± 1.21 vs. 66.8 ± 0.88, p = 0.028) after including FEV1 into the model (model 2). As shown in Table 3, the MWA was found to be lower in KOLD even after including FEV1 into the model (70.3 ± 1.25 in CODA vs. 67.1 ± 0.92 in KOLD, p = 0.04 in model 2). However, there was no difference in the ratio of MLD in CT measurements between the two cohorts.

**Table 2:** Comparison of lung function and CT-measurement in CODA and KOLD cohort studies

	Model 1			Model 2		
	CODA	KOLD		CODA	KOLD	
	LSMean ± SE	LSMean ± SE	*P*	LSMean ± SE	LSMean ± SE	*P*
CAT score	23.10 ± 2.64	11.70 ± 2.20	< .001			
Pulmonary function test						
Pre-bronchodilator FEV1 (L)	1.60 ± 0.13	1.27 ± 0.09	0.040			
Pre-bronchodilator FVC (L)	2.59 ± 0.18	2.79 ± 0.13	0.409			
Post-bronchodilator FEV1 (L)	1.72 ± 0.12	1.40 ± 0.09	0.048			
Post-bronchodilator FVC (L)	2.86 ± 0.18	2.93 ± 0.12	0.769			
Post-bronchodilator FEV1/FVC	0.59 ± 0.02	0.48 ± 0.02	0.001			
BDR, bronchodilator response	10.20 ± 2.84	12.00 ± 2.01	0.629			
CT measurement						
Mean wall area (%)	70.20 ± 1.26	67.70 ± 0.90	0.121	70.20 ± 1.21	66.80 ± 0.88	0.028
Emphysema index	6.07 ± 3.06	20.0 ± 2.21	< .001	6.12 ± 2.88	17.30 ± 2.10	0.002
Emphysema index, log-transformed/exponential	4.45 ± 1.28	16.1 ± 1.19	< .001	4.46 ± 1.27	13.60 ± 1.19	< .001
The ratio of mean lung density (expiration/inspiration)	0.95 ± 0.01	0.94 ± 0.01	0.452	0.95 ± 0.01	0.93 ± 0.01	0.077

*Model1:* adjusted for age, gender, education, BMI, smoking status, pack-year, CCI, and exacerbation

*Model2:* adjusted for Model1 plus post-bronchodilator FEV1 (L)

*CAT* COPD assessment test, *FEV1* forced expiratory volume in one second, *FVC* forced vital capacity, *LSMean* least-squared mean, *SE* standard error, *BMI* body mass index, *CCI* Charson comorbidity index

^†^LSMeans (SE) for log-transformed lung function and CT measurements were assessed by the generalized linear mixed model

**Table 3:** Comparison of lung function and CT-measurement adjusted for COPD medication and biomass exposure

	Model 1			Model 2		
	CODA	KOLD		CODA	KOLD	
	LSMean ± SE	LSMean ± SE	*P*	LSMean ± SE	LSMean ± SE	*P*
CAT score	23.60 ± 2.77	11.30 ± 2.44	< .0001			
Pulmonary function test						
Pre-bronchodilator FEV1 (L)	1.56 ± 0.12	1.27 ± 0.09	0.071			
Pre-bronchodilator FVC (L)	2.63 ± 0.19	2.84 ± 0.14	0.368			
Post-bronchodilator FEV1 (L)	1.68 ± 0.12	1.41 ± 0.09	0.087			
Post-bronchodilator FVC (L)	2.92 ± 0.18	3.00 ± 0.13	0.730			
Post-bronchodilator FEV1/FVC	0.57 ± 0.03	0.47 ± 0.02	0.003			
BDR, bronchodilator response	10.80 ± 2.99	12.40 ± 2.19	0.683			
CT measurement						
Mean wall area (%)	70.30 ± 1.29	67.90 ± 0.95	0.130	70.30 ± 1.25	67.10 ± 0.92	0.041
Emphysema index	6.24 ± 3.16	20.0 ± 2.32	0.001	6.14 ± 2.99	17.60 ± 2.22	0.002
Emphysema index, log-transformed/exponential	4.54 ± 1.29	15.22 ± 1.21	< .001	4.51 ± 1.28	13.20 ± 1.20	0.001
The ratio of mean lung density (expiration/inspiration)	0.95 ± 0.01	0.94 ± 0.01	0.372	0.95 ± 0.01	0.93 ± 0.01	0.088

*Model1:* adjusted for age, gender, education, BMI, smoking status, pack-year, CCI, exacerbation, COPD medication, and biomass exposure

*Model2:* adjusted for Model1 plus post-bronchodilator FEV1 (L)

*CAT* COPD assessment test, *FEV1* forced expiratory volume in one second, *FVC* forced vital capacity, *LSMean* least-squared mean, *SE* standard error

^†^LSMeans (SE) for log-transformed lung function and CT measurements were assessed by the generalized linear mixed model

The EI was also lower in CODA after adjusting for biomass, and COPD medication (6.24 ± 3.16 in CODA vs. 20.0 ± 2.32 in KOLD, p = 0.001, model 1 in Table 3) and was consistent with the results obtained after including FEV1 in the multivariate model (p = 0.002, model 2 in Table 3).

## Discussion

In this analysis of patients with COPD (confirmed by spirometry), MWA by CT measurements was significantly higher in the CODA cohorts which represented a rural area near cement plants. These results were consistent, even after adjusting for age, gender, educational attainment, BMI, smoking habits, CCI, spirometry parameters, history of COPD exacerbation, medication they used and biomass. Patients with COPD living in rural area showed higher incidence and events of COPD exacerbation in previous studies [20, 21]. However, the reason for the mechanism was not clearly elucidated. CT findings might provide a clue for this difference in COPD characteristics between urban and rural areas.

In this study, we measured the MWA and EI by CT scan within the two cohorts. In previous studies, CT findings showed various patterns and phenotypes for several conditions and causal materials including biomass, smoking, occupational chemicals and combustion. For instance, non-smoker and female patients with COPD had less emphysema and air-trapping, as detected by HRCT, thicker basement membranes and greater endobronchial pigmentation [12]. Non-smoker COPD subjects had lower emphysema scores and airway diseases predominant patterns in CT scan than smokers, as per a recent study [22]. The biomass also showed a variation in pattern in CT scans with lower emphysema and higher air trapping [10]. Sex difference was also suggested as mechanism of COPD in CT findings in a previous study possibly by environmental, biologic and/or genetic factors [23]. In our study, patients with COPD living in rural areas had significant CT findings with higher MWA and lower EI compared with those in urban areas after adjusting for sex, smoking habit and biomass exposure, which was previously observed to have an influence on different CT findings. In a previous study, residence in a rural area was a predictor of COPD not only for smokers but also for non-smokers [21]. The characteristics of the rural area might affect COPD mechanisms other apart from the effect of tobacco (which is a representative mechanism of urban COPD). A higher EI represented in the KOLD cohort in our study could explain the characteristics.

Participants in the CODA cohort, who lived near cement plants and were exposed to cement dust, had increased wall thickening on chest CT. In a previous study, exposure to cement dust by living or working near cement plants was associated with a higher prevalence and hospitalization of patients with COPD and who also experienced more respiratory symptoms [24, 25]. Recent studies support the fact that specific cement dust effects structural changes in CT findings. First of all, the cement dust-exposed subjects with normal spirometry demonstrated airway narrowing in the lower lobes, wall thickening at all segmental airways, a different bifurcation angle at the central airways, and a loss of airway wall elasticity at the lower lobes compared to the non-dust-exposed subjects [26]. Furthermore, dust exposure to cement was significantly associated with bronchial alterations in segmental scales rather than parenchymal changes such as emphysematous changes. As the particles from cement dust range in sizes from 0.5 to 5 um, they are usually deposited on the segmental airway [27, 28]. In the case of cigarette-smoke particles, the rate of deposition was greater in the parenchymal region than in the segmental region due to small size of the particles ranging from 0.01 to 1 um [29, 30].. Kim et al. studied the relationship between different characteristics of particles and CT findings among persons with normal spirometry in a previous study [26]. As subjects with normal spirometry results, airway structural alteration by cement dust also influenced patients with COPD who showed airway limitation in spirometry results and had a smoking intensity of more than 10PY.

Structural changes represented by wall thickness in CT have been reported in studies of other types of dust. Individuals with low FVC who were occupationally exposed to the World Trade Center disaster showed higher WA% representing proximal airway inflammation. However, there was no difference in the measurement of distal airway and emphysema compared to persons with normal spirometry [31]. The carbon black packer also showed a higher WA and lower LA on CT as reported by Xue et al. [32]. However, the shared mechanism between dust exposure and small airway thickening has not been clearly established. We added evidence that small airway wall thickening may explain the pathophysiology induced by dust exposure.

Occupational factors according to regional distinction have also influenced COPD incidence or exacerbations in previous studies. Agricultural workers who are often exposed to dust and vapors that can cause or aggravate underlying lung diseases are the most prevalent rural occupation [33, 34]. It has also been reported that agricultural work affects COPD development and aggravation of their symptoms [34–36]. Focusing on rural regions, however, the results for the effects of occupation on COPD have been inconsistent. One study found an increased risk of COPD exacerbation through a multivariate analysis, but another revealed no significant association in the adjusted model [20, 21]. Subjects in CODA cohort where about 70% of participants worked in cement factories or in farm [37] had a pathophysiology characterized by small airway thickening rather than parenchymal changes. Further long-term consequences regarding cement dust exposure on the COPD phenotype should be investigated.

Biomass exposure is another cause of COPD development which had been studied in non-smoker and female subjects [38, 39]. Biomass-related COPD had a greater reduction in mid-expiratory volume and less pronounced markers of emphysema than smoking-exposed COPD in previous studies [10, 12]. Biomass exposure is associated with greater intimal proliferation, basement membrane thickening, fewer neutrophil and greater numbers of lymphocytes and macrophages in lavage fluid. This was consistent with autopsy findings, wherein more fibrosis was found in the small airway walls and greater pigment deposition occurred within the lung parenchyma [12, 40]. In our study, subjects in both cohorts had experienced more than 10-PY of smoking and the direct exposure to charcoal and wood even though there were difference between the two groups. We found that the results were consistent after adjusting for smoking and biomass exposure. We could assume that these CT findings could be distinct characteristics of patients with COPD living in rural regions near cement plants, rather than cigarette smoking or biomass. These findings were also consistent with the fact that the incidence of COPD in rural areas has increased among both smokers and non-smokers [21].

To our knowledge, this study is the first to provide a direct comparison of the phenotype of CT findings between patients with COPD from rural and urban areas. This study could explain the different clinical characteristics of patients with COPD from a rural area. Despite the strengths of this study, there were several limitations. First, this study had a cross-sectional design, so there was no chronologic information for changes in these results due to the nature of this study design. The findings of longitudinal CT images of further studies should be followed to confirm the characteristics revealed in this study. Second, we compared two distinguished cohorts representing patients with COPD living in rural and urban areas which might have less comparability while exploring the effect of various exposures. We could not distinguish occupational exposure from regional exposure of individuals who lived within the same district. Despite this limitation, we included almost all possible confounders mediating the COPD phenotype by stepwise multivariable analysis. In addition, the intensity, duration and type of biomass which are well-known COPD phenotype factors between the two cohorts, were not compared in this study. However, as far as we included household biomass exposure, we found consistent results. Further studies regarding quantitative data of individual environmental, occupational or biomass exposure are still needed concerning their effect on CT findings, or their biological effects on COPD. Third, quantitative analysis in COPD is influenced by the CT scanner and scan parameters such as reconstruction algorithm, radiation dose, section thickness [41]. Scan parameters used in our study were designed to minimize interpatient differences, but CT scanners vary among institutions. A study showed Interscanner variability by measuring mean lung density in a humanoid phantom [42]. Therefore, EI and MLD may have affected by the difference between CT scanners in the present study.

## Conclusions

Higher MWA and lower EI were observed in the dust exposure region. These results suggest that the imaging phenotype of COPD is influenced by environmental exposure.

## Data Availability

The datasets used for the current study are available from the corresponding author on reasonable request.

## Abbreviations

COPD: Chronic obstructive lung disease
AE: Acute exacerbation
CT: Computed tomography
CODA: COPD in dusty area
KOLD: Korean obstructive lung disease
PFT: Pulmonary function test
MLD: Mean lung density
WA: Wall area
LA: Lumen area
WA%: Wall area percent
MWA %: Mean wall area percent
EI: CT-emphysema index
